# Reaffirming DEI as a central value of JCM

**DOI:** 10.1128/jcm.00372-25

**Published:** 2025-03-31

**Authors:** Romney M. Humphries, Alexander J. McAdam

**Affiliations:** 1 Vanderbilt University Medical Center 12328, Nashville, Tennessee, USA; 2 Department of Laboratory Medicine, Boston Children's Hospital 1862, Boston, Massachusetts, USA

**Keywords:** clinical microbiology

## EDITORIAL

For half a century, the *Journal of Clinical Microbiology* (JCM) has been the home for publications in our field. Moreover, we see JCM as strengthening the clinical microbiology community by providing a venue for educational materials, and through the letters, commentaries, and editorials that engage with the questions and challenges we face as a field. As we navigate the rapid changes in U.S. federal policies, some have raised understandable concerns about the impartiality of scientific publications supported by organizations funded through federal grants. Now, more than ever, it is important for us to reaffirm our commitment to ethical scientific publishing and our vision for JCM over the next 5 years. We will continue to publish robust, significant, and original scientific articles following Committee on Publication Ethics (COPE) guidelines for ethical standards in scholarly research and publication. At the same time, we will renew our strong focus on diversity, equity, and inclusion.

Diversity, equity, and inclusion have long been a mission of JCM and of ASM Journals. There are many important reasons for this focus within JCM.

First, JCM is a medical journal. All individuals deserve equitable and accessible healthcare, and a focus on the role of diagnostics to this end is an important research priority for our field. Disparities in testing access have been shown to negatively impact marginalized people. For example, COVID-19 testing sites in the United States underserved the racial and ethnic populations who bore a disproportionate burden of COVID-19 cases and deaths (1). Research surrounding increased access, building on the precedent and success of providing HIV diagnostic testing in alternative settings (2), can drive alternative test delivery models, and elevate the care of all patients afflicted by microbial diseases.

We will also work toward ensuring that the performance of clinical microbiology tests in diverse populations is understood. Diagnostic testing for infectious diseases is complex, in part because pathogen distributions vary across race, sex, and age, as do biological markers of infectious diseases, such as sepsis. Ensuring the representation of diverse populations in studies that evaluate next-generation tests, artificial intelligence diagnostic models, and prognostic tools is crucial to the development of tools that meet the needs of all patients (3). Significant research is needed to ensure testing accurately predicts disease for specific populations, for example, bacterial vaginosis tests developed for cisgender women do not meet the needs of transmasculine people (4). Inclusion of our diverse populations in such studies can be challenging, as some individuals do not trust the medical scientific complex due to historic and recent injustices (5). As such, special emphasis must be made to ensure that studies of these tests and systems include these populations. The work we do in this space is important: articles published by JCM are read by policymakers and people developing clinical standards worldwide, and so they have the potential to significantly impact the discourse around disparities in testing and its impact on health.

Second, infectious diseases are not an affliction that only impacts the global north. However, U.S. clinical microbiologists from academic medical centers have been disproportionately represented on our editorial board and among our Editors. The inclusion of Editors from geographically diverse regions who are familiar with different contexts for diagnostic testing is an important component of our vision for a more inclusive and diverse JCM. Understanding what is happening in countries outside the United States, and indeed in under-represented regions inside the United States, enables us to adapt to arising infectious disease threats, alternative approaches, and cost-effective care.

Third, publication in journals like JCM remains the primary means of scholarly communication and an indicator of scientific merit. And yet, few of the members of our community are funded, publishing quality, pragmatic data that are invaluable for the field using shoestring budgets. We benefit from this work as a field—and publication fees should not be a barrier to the promotion of important, unfunded studies. Similarly, access to content behind paywalls should not be a barrier to the publication of solid science.

Finally, we must recognize we live in a society where gender, race, religion, and sexual orientation continue to be barriers to opportunity. Biases underlying this are deep, and too often minority scientists have experienced unequal burdens and limited opportunities to focus on academic growth. This erodes their sense of worth and leads them to question the value of their contributions (6, 7). As leaders in our field, it is incumbent on us to provide fair and just opportunities to learn, contribute, and publish to all members of our community. This cannot happen organically and requires dedication to DEI.

Commitment to these principles requires tangible action:

1. We will recruit an Editor to focus on DEI for the Journal, who can solicit content and highlight the importance of DEI in clinical microbiology. There is a tremendous opportunity for research on diagnostic access, epidemiology, and diseases that impact underrepresented groups in clinical microbiology. JCM can and should be the home for these publications.
2. We will explore avenues to ensure that the publication of good science in JCM is equitable to all authors, supporting and promoting researchers based on their science.
3. We will continue to explore mechanisms of equitable access to JCM content, building on the existing strong infrastructure for open-access articles across ASM Journals. More than this, we will diversify our ways of communicating to ensure that the science published in the journal is accessible, including through podcasts, lay summaries, editorial features, and other non-traditional content
4. Finally, we will build on our mentorship programs, providing education and opportunities within the journal for all clinical microbiologists, with a focus on junior clinical microbiologists and those historically underrepresented within JCM.

For many years, JCM has set the standard for diagnostic innovation in clinical microbiology, introducing controversial topics to the forefront of scientific discourse, and it has provided a platform for commentary and debate. Regardless of the political landscape, JCM will continue to be the academic home for clinical microbiology. We have benefited from a diverse and strong leadership that includes many individuals from groups traditionally underrepresented in the sciences. The success of JCM requires building on this diverse expertise across our Editors, Editorial Board, and contributors to pave the pathway for the Journal in the coming years. This effort strengthens JCM and our impact on the global scientific landscape. More than that, it enriches our field with strong leaders who can support the microbial sciences and clinical care for all populations.

This editorial reflects the views of the authors in their positions as editors of the J*ournal of Clinical Microbiology*.
